# A Controlled Approach to the Emotional Dilution of the Stroop Effect

**DOI:** 10.1371/journal.pone.0080141

**Published:** 2013-11-06

**Authors:** Kathryn Fackrell, Mark Edmondson-Jones, Deborah A. Hall

**Affiliations:** 1 Division of Psychology, Nottingham Trent University, Nottingham, United Kingdom; 2 NIHR Nottingham Hearing Biomedical Research Unit, University of Nottingham, Nottingham, United Kingdom; Baycrest Hospital, Canada

## Abstract

We re-examined a modified emotional Stroop task that included an additional colour-word alongside the emotional word, providing the response conflict of the traditional Stroop task. Negative emotionally salient (i.e. unpleasant’) words are claimed to capture attention, producing a smaller Stroop effect for negative words compared to neutral words; this phenomenon is called the emotional dilution of the Stroop effect. To address previous limitations, this study compared negative words with lexically matched neutral words in a powered sample of 45 participants. Results demonstrated an emotional Stroop effect (slower colour-naming responses for negative words) and a traditional Stroop effect but not an emotional dilution of the Stroop effect. This finding is at odds with claims that other processing resources are diminished through the failure to disengage attention from emotional information. No matter how attention towards emotional information builds up over time, our findings indicate that attentional resources are not fully captured by negative words.

## Introduction

A popular paradigm widely used to investigate the role of emotion on attention and cognition is the emotional Stroop. This is a variant of the traditional colour-word Stroop paradigm [1,2]. The traditional Stroop task involves naming colour words presented either in a congruent condition (the word “red” printed in red ink) or an incongruent condition (the word “green” printed in red ink). The Stroop effect (SE) refers to the performance cost arising from the additional requirements to inhibit the pre-potent response to the written word in the incongruent condition. This performance cost is measured by subtracting the incongruent condition from the congruent. In the emotional Stroop task, participants are required to respond to the ink colour of various written words including those that are emotionally salient (positive salience e.g. “beauty” and negative salience e.g. “slum”), whilst ignoring the semantic meaning of the words. Typically, response times (RTs) are longer for emotional words than emotionally neutral words; the emotional Stroop effect (ESE). This effect has been applied to explain attentional bias in clinical populations with an emotional disorder when the emotional connotations of the words correspond with the concerns surrounding the disorder, i.e. threat-related words [3–5]. For example, patients who experience tinnitus exhibit faster responses to tinnitus-related words (piercing, buzz and howling) than the neutral words (cow, goat and crop) [6]. 

For the ESE, the difference in colour-naming RT’s for emotional words and for neutral words establishes the size of the interference [7]. The performance cost here is typically assumed to reflect allocation of attention towards the negative emotional relevance of the word meaning. While it is uncertain whether it is the negativity, the threat or the arousal of emotional stimuli that increase RTs, this phenomenon is believed to be an indicator of emotional attention bias [2], or attentional capture and failure to disengage [8]. In terms of attention bias, negative words might reflect a fast and automatic effect which taps the influence of emotional arousal on the current trial, diverting attention away from the colour-name which is the task relevant feature. In addition attention bias also includes a slow process that builds up over time from trial to trial, indicating a warning system which monitors the external environment in the presence of threat. In terms of the failure to disengage, emotional words hold attention for longer, causing a freezing effect with problems disengaging attention from the word and focusing on the colour dimension. While the two paradigms have evolved with different purposes in mind, it is nevertheless true to say that one common interpretation of both the traditional colour-word Stroop and the emotional Stroop involves the notion that people cannot ignore the semantic meaning of isolated words and performance is compromised by processing the task-irrelevant stimulus content [7,9]. 

Controversy surrounds the ESE with three concerns focusing around the conceptual association to the SE and the methodological similarities and differences between these two paradigms [1,10]. Here we consider each one briefly in turn. 

### I: The emotional Stroop task lacks a conflicting pre-potent response

One issue concerns the degree to which the traditional and emotional Stroop tasks assess the same cognitive function, i.e. the cognitive conflict to resolve the incongruence between the stimulus target and the pre-potent response [10]. The emotional Stroop paradigm does not involve any such conflict; there are no congruent or incongruent items [11]. For example, the neutral word “hat” printed in red ink is no more or less congruent or incongruent than the emotional word “wicked” printed in blue ink. The ink colour and the emotional word meaning do not lie on the same dimension and therefore lack the logical response conflict between attributes that the traditional Stroop performance hinges on (e.g. [12]). 

### II: The traditional Stroop effect incorporates a supplementary role of facilitation

According to the logic of cognitive subtraction, the SE includes components not only relating to the inhibition of response (incongruent) but also facilitation of response processing (congruent). Within the congruent condition both the form of the colour word and the ink colour presented lead to the same response. The colour-word red and the ink colour red represented the same semantic concept and hence are processed faster, enhancing the performance. Consequently, the measure of the SE reflects a sum of inhibition and facilitation [13], although we do acknowledge that some Stroop tasks have applied a neutral condition to separate out these effects (e.g. [6,14,15]). The emotional Stroop does not involve facilitation from the influence of congruency between stimulus and response because, as mentioned above, there is no semantic relationship between the emotional word and the colour presented. 

### III: The emotional Stroop has been contaminated by uncontrolled lexical features

The ESE has been claimed a misnomer, since the effect potentially measures distinct dimensions compared to the SE [2,11]. The traditional Stroop task incorporates four colour-name words that are used in both congruent and incongruent conditions. Here lexical control is implicit because the words are identical across conditions. This is not the case in the emotional Stroop task. The words items represented across the negative and neutral word conditions are never the same. Hence lexical control becomes an important factor in experimental design [7]. Indeed Larsen and colleagues go as far as to state: “If researchers want to infer that any slowdown in response latencies to negative words (compared with neutral words) is due to the emotional content of the words, then it is absolutely crucial that the emotional and neutral words be matched on lexical features known to influence word recognition.” pp 67. They claim that interference effects reported in the literature could reflect lexical differences rather than differences in conflicting processes and emotional “capture”. For example, evidence has shown that colour-naming of low frequency words is slower than high frequency words [9]. Longer words and low neighbourhood words take longer to process than shorter, high neighbourhood words and hence would cause more interference to task-relevant information [16,17]. In their systematic analysis of 32 investigations into the ESE, Larsen et al. [7] analysed 1,033 words in different valence word categories, and found that majority of previous studies were unbalanced for word frequency (N = 21 (66%)), word length (N = 16 (50%)) and neighbourhood density (N = 17 (53%)). Valence refers to ratings of pleasure: unpleasant to pleasant. Emotionally negative, unpleasant words were found to be rarer, longer and have lower neighbourhood density than both neutral and positive words. This potentially corrupts the interference, making the results uncertain. 

Arousal could also impact on RTs for word recognition, although the evidence for this impact is rather mixed. Kousta, Vinson and Vigliocco’s [18] findings suggest that emotional valence, not arousal, predicted lexical decisions to emotional words, whilst Larsen, Mercer, Balota and Strube [19] found that it was arousal that increased recognition times for emotionally negative words. Although the emotional Stroop paradigm measures somewhat different cognitive processes from that of word recognition, this evidence does suggest that arousal could potentially impact on attentional bias. For the emotional Stroop paradigm, some researchers have suggested that arousal, not valence, drives the bias towards the emotional words, and therefore the ESE [19,20]. Some previous emotional Stroop studies have selected negative words with higher arousal ratings than neutral words [21,22], which makes it difficult to attribute the cause of the ESE and to disentangle the effects of arousal on the ESE. Although most research has started to address some of the criticisms made by Larson et al.[7], many studies still overlook the need to control for lexical features [23]. This is especially true for neighbourhood density which is generally not reported [22,24,25]. 

**Figure 1:**
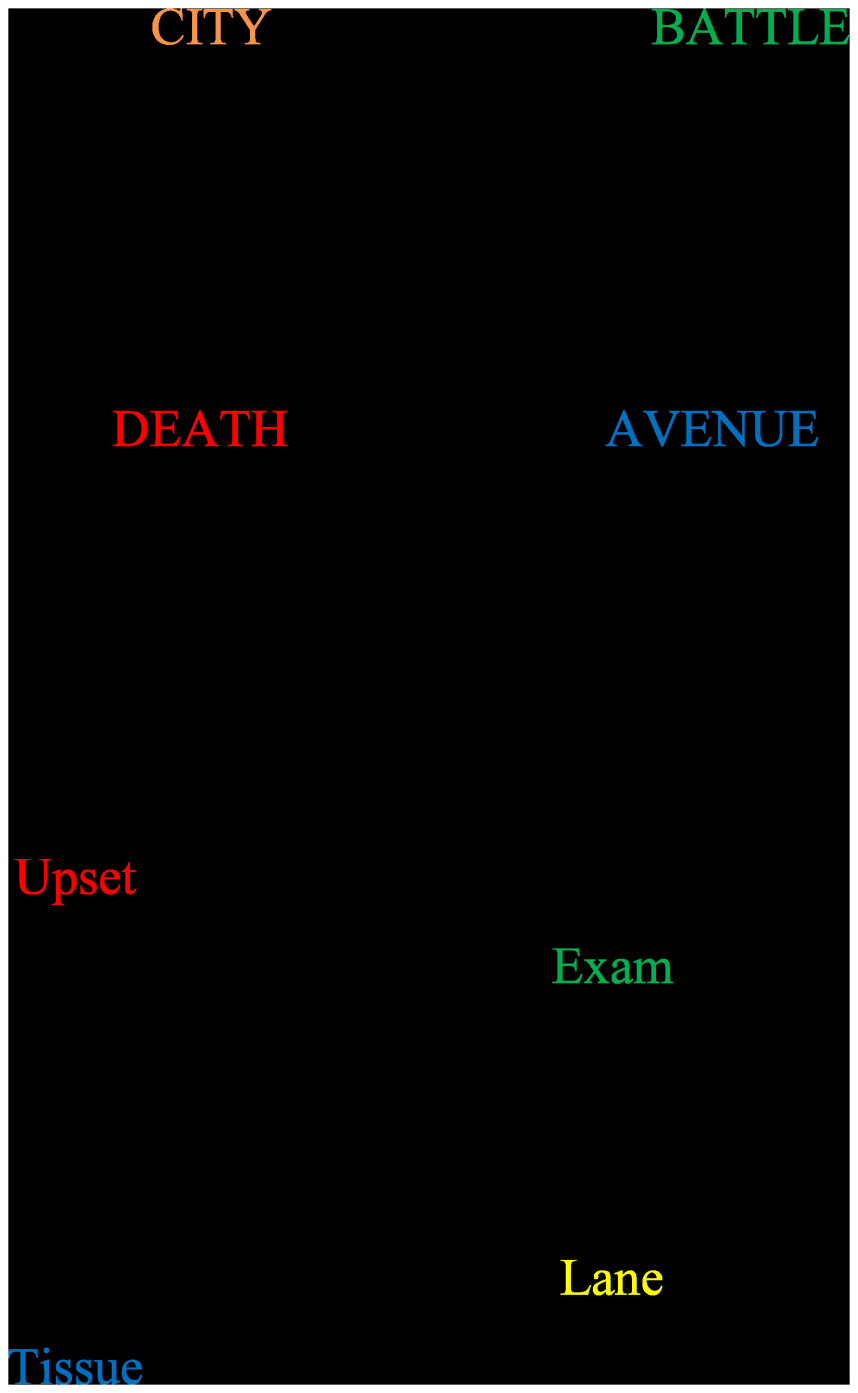
Examples of screen display. (a) shows examples of the words (negative and neutral), colours and possible format used by Chajut et al (2010) [26]. (b) shows examples of the format used in this current study; the presentation location of the words (including counterbalance), the words (negative and neutral-matched) and colours.

In 2010, a modified paradigm within emotional Stroop was reported [26] that addressed concerns I and II. The paradigm, with the inclusion of an additional colour-word, combines response conflict with colour-word incongruence as well as facilitation with colour-word congruence (Figure 1). In this version, the colour-name word (i.e. the word “red”, presented in black ink) and a colour-carrier word either emotionally negative (i.e. “injury” presented in red ink) or emotionally neutral (“city” presented in yellow ink) were presented side by side on the screen (Figure 1). The ink of the colour-carrier word either denoted the same meaning as the colour-name word (congruent) or a different meaning (incongruent) (Figure 1). Consequently, within each carrier-word condition (i.e. emotionally negative or emotionally neutral) the difference between congruent and incongruent condition can be obtained, demonstrating the traditional SE. Participants were required to respond to the ink colour of the colour-carrier word as quickly as possible, whilst ignoring the meaning of the word. The reported finding was that there was a smaller response cost (i.e. a smaller difference between congruent and incongruent conditions) for emotional words compared to neutral words. This phenomenon was called the “emotional dilution of the SE”. The inclusion of the additional colour-word reduces the SE for emotional words. The interpretation of such an effect was that the emotionally negative words capture attentional processing resources over and above that which is required to resolve the cognitive conflict in the incongruent conditions. However, this study was somewhat limited by: i) an ESE was not reported, failing to replicate previous work, ii) a strong interpretation of the emotional dilution of the SE findings considering the lack of an overall effect of emotion on RTs iii) highly variable effect sizes across their two experiments, iv) relatively low statistical power (e.g. 15 participants in Experiment 1, analysis of summary data with only 13 degrees of freedom), and v) the poor control over lexical features.

The position on the screen where the words are presented is also worth considering because words presented within the left visual field project to the right hemisphere, which plays a dominant role in processing of emotional words particularly negative emotions [27]. Borkenau and Mauer [28] have shown that participants take longer to name the colour of negative words presented in the left visual field than those presented in the right, reinforcing the need to avoid hemispheric processing asymmetries. The presentation of emotional words, side by side, to the left and right of fixation could influence the attentional “capture”. Within Chajut et al’s [26] modified emotional Stroop paradigm there are a number of problems concerning word presentation. It is unclear whether the emotional words were presented to right or left of the fixation and there was no report of counterbalancing of the word position. 

The present study re-examined the modified emotional Stroop paradigm. Our study design was based on the paradigm reported by Chajut et al. [26], but used a powered sample and carefully controlled for lexical characteristics and word position. We addressed two questions:

i) Do the Stroop effect (SE), an emotional Stroop effect (ESE), and a dilution of the emotional Stroop effect remain when stimulus selection carefully controls for lexical factors?
ii) How does non-matching the words in the neutral comparison condition impact on the above?

## Methods

### Participants and sample size calculation

A sample size estimate was based on the previous published data reported by Chajut et al. [26]. To be conservative, we calculated the required power by using the smaller Stroop effect of Experiment 2. This was combined with the published standard deviations (from Experiment 1). Furthermore to maximise the sample size requirements, we made the assumption that the Stroop effects for the neutral and emotionally negative words were not expected to be correlated. This conservative approach would give us the largest sample size required, ensuring that our study was more than sufficiently powered on the basis of the data previously reported. Based on this information, a sample of at least 27 participants is required to detect both main effects (SE, ESE) and interaction (dilution effect) with an overall power of 80%. 

An opportunity sample of 45 students and staff (10 men and 35 women, mean age = 24 years, SD = 8) were recruited from Nottingham Trent University and the University of Nottingham. All participants reported unimpaired colour vision and were native English speakers. Students were offered course credits as part of the university scheme. 

### Ethics Statement

All participants were provided with a participant information sheet, and given the opportunely to raise any questions or concerns. Following this, informed, signed written consent was obtained from the participants before commencing the experiment. Ethical approval was granted for the entirety of this study including the consent procedure by the Social Sciences Research Ethics committee, Nottingham Trent University (Date of SREC meeting: 29/11/2011).

### Materials

The study used 192 words (see Tables S1 - S3), with affective valence of the carrier (emotionally negative (i.e. unpleasant), ‘neutral-matched’ and ‘neutral-nonmatched’ words), neighbourhood density (zero and high) and colour-word congruence (congruent and incongruent) as factors, in a partial factorial design. In terms of controlling lexical features, the neutral-matched words were explicitly controlled in order to match the lexical features (word frequency, length, arousal and neighbourhood) of the emotionally negative words. To facilitate matching on key lexical dimensions known to affect RTs, arousal and valence ratings were taken from the Affective Norms for English Words (ANEW) database [29] which provides a set of normative ratings for valence and arousal on 1011 words that were collected via a uniform procedure. Neighbourhood density and frequency ratings were obtained from the English Lexicon Project (ELP) [30], as recommended by Larsen et al. [7]. Ratings of emotional valence provided a quantitative marker for classifying word categories [18,22,31], with low valence words (score <4.0) denoting the negative ‘unpleasant’ category and mid-range valence words (score 4.0 - 6.5) denoting the neutral category (Table 1). Negative and ‘neutral-matched’ conditions were matched (p> 0.05) for word frequency, word length and arousal (Table 1), although this did mean that the neutral-matched condition had a higher mean arousal rating than previous studies [21,22]. The ‘neutral-nonmatched’ condition was not explicitly matched to either negative or ‘neutral-matched’ conditions. The purpose here was to address the main question by determining the importance of lexical control on the overall interpretation of the ESE. As in Chajut et al. [26],, the neutral-nonmatched condition had a higher frequency and were less arousing than the negative condition (p< 0.05), but did not significantly differ in length (p> 0.05). Neighbourhood density was manipulated across negative and neutral-matched word conditions. Each condition had two levels of neighbourhood density; high neighbourhood ranged from N = 5 to 23, low neighbourhood N was zero. Neighbourhood density was not explicitly manipulated across neutral-nonmatched words, but nevertheless did not significantly differ from the other conditions (F (2,189) = 0.340, p= 0.713), see Table 1. 

**Table 1:** Characteristics of word battery.

**Emotional carrier**	**Valence**	**Neighbourhood density**	**Word frequency**		**Length**	**Arousal**
			**HAL norms**	**Log transformation**		
**Negative**	2.70 (0.65)	4.63 (5.63)	11527.87 (16552.58)	8.13 (1.79)	5.11 (0.74)	5.27 (0.99)
**Neutral-matched**	5.58 (0.59)	5.11 (6.56)	12550.61 (17616.97)	8.55 (1.51)	5.11 (0.08)	5.06 (0.85)
**Neutral-nonmatched**	5.46 (0.61)	4.25 (5.5)	31598.47 (76320.90)*	8.87 (1.76)*	5.20 (1.80)	4.53 (0.59)*

Data derived from ANEW database and ELP database for valence and neighbourhood density factors and the controlled lexical features of the words. Table reports the means (standard deviations). HAL: Hyperspace Analogue to Language norms [36]. *p<.05 significant difference between neutral-nonmatched and negative condition.

### Procedure

On each trial, to avoid potential problems of hemispheric processing asymmetries, the pair of words was presented above and below a central fixation. The words were presented on a light grey background in the centre of the 14 inch monitor in Arial 28 font, using SuperLab (V4.5). One word was the carrier (e.g. “battle”) and the other was a colour name (e.g. “red”). The carrier word was presented in red, green, yellow or blue coloured font. The colour-name word was always presented in black font. Word position was fully counter-balanced across participants such that if the carrier word (e.g. “slum”) appeared above fixation for one participant, it would appear below for another (see Figure 1). On half the trials, the carrier word was presented in the same colour font (congruent) as the colour-name word. On half the trials, the carrier word was presented in a different colour font (incongruent) as the colour-name word. 

For familiarisation of the paradigm, participants carried out an initial practice block using a set of carrier nonwords (‘Vnvwy’) (sourced at MCword database [32]). Participants were instructed to fixate on the centre and to identify the name of the coloured font of the carrier word (i.e. the nonblack font). Responses were made using a button box labelled using the letters R, B, G, and Y. Word pairs remained visible until a response was made. RTs were measured as the time from word stimuli appearance on the screen until the button press.

An epoch presentation method in which a series of trials were of the same carrier-word condition (i.e. negative words epoch) was chosen over a mixed presentation method in which conditions were randomised in an individual trial-by-trial basis. Evidence indicates that the epoch presentation method is more likely to elicit larger RTs and ESE in healthy individuals [20,22,24]. The experiment comprised two runs each made up of 12 epochs (4 of each carrier condition), with 8 trials in each epoch. The fixation point appeared after each stimulus set and was displayed for 1500 ms before the next stimulus trial. Each epoch was separated by an extended fixation point (5 s), thereby reducing the likelihood of the slow-rate interference from emotional words carrying over to neutral words [2]. Epoch order was pseudo-randomised (i.e. ABCBAC), avoiding the sequential epochs of the same carrier-type therefore reducing automatic responses [20]. Each carrier word was presented only once and the order was randomised across participants. 

## Results

Overall error rate was 5.1%, and all error trials were excluded from further analysis. Errors did not significantly differ across conditions (p>0.05). To optimise statistical power, RT analysis was conducted using individual trial data within a generalised linear mixed model. Modelling assumed a gamma distribution and log link function. Outliers with RTs above 2 s were excluded and this accounted for a small proportion of the data (1.5%), excluding the error trials. For the negative condition there were 62 outliers, 36 for the neutral-matched condition and 30 for the neutral-nonmatched condition. 

In order to systematically investigate the data, the analysis was divided into two subsections. The first analysis used data from the negative and neutral-matched conditions, thus considering conditions in which neighbourhood factor was carefully controlled. The second was conducted on the whole dataset which enabled specific planned comparisons between the three carrier-word valence conditions (negative, neutral-matched, neutral-nonmatched), but collapsed the neighbourhood factor and potentially lost some of the lexical control. All modelling accounted for any influence of colour (blue, green, red or yellow), word position (above or below) and the different grammatical categories (nouns, verbs and adjectives) on the three main factors of interest (i.e. carrier-word valence, colour-word congruence and neighbourhood density). However, we did not specifically examine the significance of these effects, nor any interactions with the three main factors, because this was not one of our study objectives. 

### 1): Effects of controlling the stimulus selection on the ESE

This section of analysis is concerned with performance on the negative and neutral-matched conditions when words had been carefully controlled for the following lexical factors; word length, frequency, arousal and neighbourhood density. The model specified carrier-word valence (negative or neutral-matched), colour-word congruence (congruent or incongruent), and neighbourhood density (low or high) as factors. 

Results confirmed a significant SE (F (1, 5.41) = 12.03, p = 0.001), with faster colour-naming RTs in the congruent condition compared to the incongruent condition. In addition, there was a significant ESE (F (1, 5.41) = 4.99, p= 0.033), with colour-naming RTs being slower for the negative condition than the neutral-matched condition (Table 2). There was no significant interaction between these two factors, congruence did not interact with carrier-word valence (F (1, 5.41) = 0.20, p = 0.68). This null finding goes against the prediction of an emotional dilution of the SE, but unlike Chajut et al [26], we were able to replicate both SE and ESE

**Table 2:** Response times.

**Carrier-word valence**	**Congruence**	**Mean RTs (ms)**	**SD**	**SE**	**ESE (negative – neutral)**
**Negative**	Congruent	825.01	307.39	37.06	―
	Incongruent	862.06	318.88		
**Neutral-matched**	Congruent	824.64	305.21	31.15	9.67
	Incongruent	843.09	312.89		
**Neutral-nonmatched**	Congruent	802.43	289.66	18.45	25.56
	Incongruent	833.58	314.80		

The mean RTs and standard deviations (SD) for the negative, neutral-matched and neutral-nonmatched carrier-word conditions, split according to colour-word congruence. The size of the SE and ESE is also reported.

Perhaps surprisingly, we did not find any difference in RTs between low (mean: 830ms) and high neighbourhood density words (mean: 830ms) (F (1, 5.41) =0.01, p = 0.944). No significant interactions were found between neighbourhood density and carrier-word valence (F (1, 5.41) = 0.23, p = 0.624), or congruence (F (1, 5.41) = 1.26, p = 0.245). There was no significant three-way interaction (F (1, 5.41) = 0.11, p = 0.731). The implication is that neighbourhood density appeared to have no adverse effect on the ESE. This is somewhat consistent with Larsen et al.’s (2006) original proposal that the effect of neighbourhood density on the ESE might be small. For example, neighbourhood density was found to be only weakly related to longer colour-naming responses [19].

### 2): Consequences of non-matching

In order to establish the effect of not controlling for some of the potentially important lexical factors, this analysis included the neutral-nonmatched condition in a re-examination of the SE, ESE and the emotional dilution of the SE (see Table 2). The model incorporated colour-word congruence (congruent or incongruent) and carrier-word valence (negative, neutral-matched or neutral-nonmatched) as factors. Significant main effects for congruence (F (1, 8.13) = 18.53, p< 0.0001) and carrier-word valence (F (1, 8.13) = 4.35, p= 0.039) again confirmed both a SE and ESE, respectively. We observed a marginally significant difference between the neutral-matched and neutral-nonmatched words (p = 0.056), with slightly faster RTs to the neutral-nonmatched words (Table 2). The direction of this difference could bias findings towards an inflated ESE, if comparing the negative condition with a neutral-nonmatched condition. In light of this result, we would agree with Larsen et al. [7] in recommending lexical control in study design. 

Crucially we did not find any significant interaction between carrier-word valence and colour-word congruence (F (2, 8.13) = 0.16, p = 0.844). This null result indicates that the size of the SE was equivalent for all the carrier-word valence conditions and no dilution occurred (Table 2). 

## Discussion

In this modified emotional Stroop task, including an additional task-irrelevant stimulus (colour-word) provides the response conflict on which the traditional Stroop task is based. This approach is therefore considered to be an appropriate way to assess the mechanisms of selective attention for emotion [33]. The current study extended this work in a powered study design. We examined the impact of lexical control on the magnitude of the ESE and also sought replication of the emotional dilution of the SE. Overall, we have established that the SE and the ESE both remain after stimulus matching but our paradigm did not produce an emotional dilution of the SE and showed a marginal effect of stimulus matching. We suggest that the emotional dilution of the SE obtained by Chajut et al. [26] might be more reflective of low statistical power rather than lack of lexical control. In our study, performance on the negative word condition was sensitive to concomitant stimulus conflict. A reasonable explanation for our non-significant interaction between carrier-word valence and colour-word congruence suggests that attentional resources are not fully captured by the emotionally negative word and that some residual resources are therefore available for deployment across simultaneously presented stimuli or tasks. We recommend that future research should implement this modified emotional Stroop paradigm (with control for word position and lexical factors), when investigating the effect of emotion on cognition. 

Previous research indicates that the ESE might be reduced or even disappear when important lexical features are controlled [7,34]. The current study investigated this issue. Findings showed a robust ESE both for the neutral-matched and the neutral-nonmatched baseline controls, contradicting this prediction. Nevertheless, there is still a case to be made for controlling lexical features especially since we observed a marginally significant difference between the neutral-matched and neutral-nonmatched with the direction of this difference potentially providing a bias towards an inflated ESE when using a neutral-nonmatched word comparison. Use of a neutral-matched comparison ensures that any interference effects can be more confidently attributed to the emotional connotations of the words diverting attention from task relevant stimuli because the incidental impact of lexical differences has been ruled out [35]. 

Although not the main research objective of the current study, it is worth briefly considering whether the present data support any of the three main classes of explanation that have been proposed to account for the ESE. These are; i) freezing of processing pathways, with reduced ability to disengage attention from the negative words, thus drawing resources away from colour-naming processes [8,33], ii) fast automatic attentional bias towards the negative words, again with resources being drawn away from colour-naming processes [12], and/or iii) a generalised slow bias due to screening for threat [2]. Our results did not support the first explanation because participants were no less vulnerable to the interference (response conflict) caused by the incongruent colour-word stimulus trials on negative words, than on neutral words. The second and third explanations cannot be directly teased apart since a mixed design (using emotional and neutral stimuli) with large inter-trial intervals would be needed to separate out the contributions from fast and slow effects [1,2]. 

In conclusion, the modified emotional Stroop task includes key elements of facilitation and inhibition, demonstrating a clear association to the traditional Stroop task. Careful control over word position and lexical features within this study have ruled out the incidental impact of lexical differences. The interference effects found can be attributed to the emotional properties of the words diverting attention. Our findings show that with lexical controls in place not only did the ESE occur but so did the SE. This implies that, although emotionally negative words ‘captured’ attention, performance was still susceptible to concomitant colour-word congruence, no emotional dilution of the SE occurred. Whilst these findings potentially rule out claims that additional resources are diminished through the failure to disengage attention from emotional information, the differential contributions of fast automatic bias and the generalised slow bias are less clear. Consequently, we conclude that emotional information does not capture attention as fully as first presumed, there is still an awareness of additional stimuli. 

## Supporting Information

Table S1 **Negative word characteristics.** The negative words individual data for the valence, neighbourhood density, word frequency, arousal, and length. Also show is the ANEW code as per the ANEW guidelines set by Bradley & Lang (2010) [29]. Organised according to neighbourhood density followed by length of the word (zero to high neighbourhood; short to long).
(DOCX)

Table S2 **Neutral-matched word characteristics.** The neutral-matched words individual data for the valence, neighbourhood density, word frequency, arousal, and length. Also show is the ANEW code as per the ANEW guidelines set by Bradley & Lang (2010) [29]. Organised according to neighbourhood density followed by length of the word (zero to high neighbourhood; short to long).
(DOCX)

Table S3 **Neutral-nomatched word characteristics.** The neutral-nonmatched words individual data for the valence, neighbourhood density, word frequency, arousal, and length. Also show is the ANEW code as per the ANEW guidelines set by Bradley & Lang (2010) [29]. Organised according to the length of words (short to long).
(DOCX)
