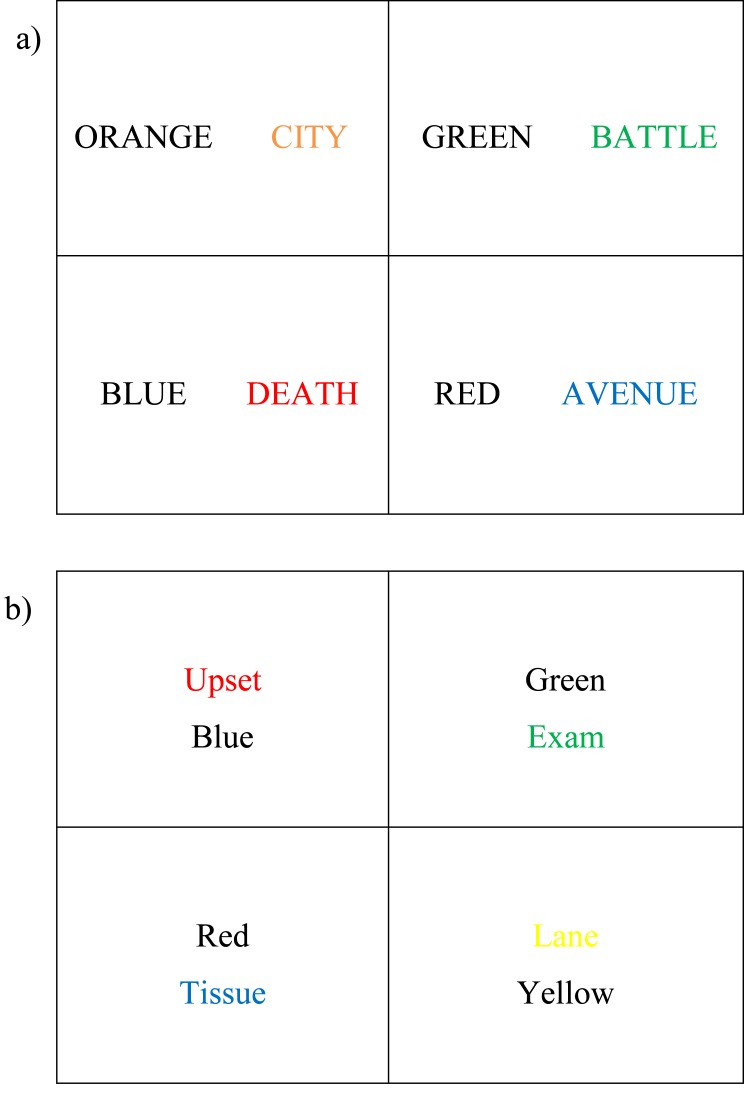# Correction: A Controlled Approach to the Emotional Dilution of the Stroop Effect

**DOI:** 10.1371/annotation/3213e7fb-c577-45c0-81c5-89b60331dbba

**Published:** 2013-12-17

**Authors:** Kathryn Fackrell, Mark Edmondson-Jones, Deborah A. Hall

There were multiple errors introduced in Figure 1 during the preparation of this manuscript for publication. Please view the correct Figure 1 here: 

**Figure:**