# Supplementary material for: A Controlled Approach to the Emotional Dilution of the Stroop Effect
Source: PLoS One. 2013 Nov 6;8(11):e80141. doi: 10.1371/journal.pone.0080141 (PMC3819280; doi:10.1371/journal.pone.0080141)
Supplement: Table S1 — Negative word characteristics. The negative words individual data for the valence, neighbourhood density, word frequency, arousal, and length. Also show is the ANEW code as per the ANEW guidelines set by Bradley & Lang (2010) [29]. Organised according to neighbourhood density followed by length of the word (zero to high neighbourhood; short to long). (DOCX) [file pone.0080141.s001.docx]

**Table S1.**

|  |  |  | Word frequency | |  |  |  |
| --- | --- | --- | --- | --- | --- | --- | --- |
| Words | Valence | Neighbourhood density | HAL norms | LOG transform | Arousal | Length | ANEW code |
| Liar | 2.41 | 0 | 6138 | 8.72 | 6.38 | 4 | 1784 |
| Ugly | 2.43 | 0 | 11466 | 9.35 | 5.38 | 4 | 460 |
| Odor | 2.52 | 0 | 1395 | 7.24 | 5.13 | 4 | 1909 |
| Exam | 2.76 | 0 | 4218 | 8.35 | 7.03 | 4 | 1510 |
| Evil | 3.23 | 0 | 43736 | 10.69 | 6.39 | 4 | 741 |
| Envy | 3.41 | 0 | 2556 | 7.85 | 5.5 | 4 | 1497 |
| Mourn | 1.8 | 0 | 571 | 6.35 | 5.87 | 5 | 1870 |
| Upset | 2 | 0 | 11355 | 9.34 | 5.86 | 5 | 465 |
| Vomit | 2.06 | 0 | 1313 | 7.18 | 5.75 | 5 | 481 |
| Agony | 2.43 | 0 | 2245 | 7.72 | 6.06 | 5 | 10 |
| Theft | 2.67 | 0 | 4875 | 8.49 | 5.96 | 5 | 2336 |
| Argue | 2.83 | 0 | 20857 | 9.95 | 6.07 | 5 | 1102 |
| Mucus | 3.34 | 0 | 419 | 6.04 | 3.41 | 5 | 886 |
| Doubt | 3.43 | 0 | 48652 | 10.79 | 4.5 | 5 | 1442 |
| Wrath | 3.47 | 0 | 5643 | 8.64 | 5.6 | 5 | 2470 |
| Avoid | 3.62 | 0 | 45327 | 10.72 | 5.04 | 5 | 1124 |
| Rigid | 3.66 | 0 | 3135 | 8.05 | 4.66 | 5 | 963 |
| Erode | 3.67 | 0 | 368 | 5.91 | 5.17 | 5 | 1502 |
| Morgue | 1.92 | 0 | 444 | 6.1 | 4.84 | 6 | 285 |
| Stress | 2.09 | 0 | 12215 | 9.41 | 7.45 | 6 | 413 |
| Corpse | 2.18 | 0 | 1985 | 7.59 | 4.74 | 6 | 86 |
| Stupid | 2.31 | 0 | 45414 | 10.72 | 4.72 | 6 | 415 |
| Sewage | 2.61 | 0 | 791 | 6.67 | 4.57 | 6 | 2165 |
| Flabby | 2.66 | 0 | 180 | 5.19 | 4.82 | 6 | 167 |
| Fungus | 3.06 | 0 | 885 | 6.79 | 4.68 | 6 | 179 |
| Ignore | 3.15 | 0 | 27790 | 10.23 | 4.67 | 6 | 1711 |
| Impair | 3.18 | 0 | 441 | 6.09 | 4.04 | 6 | 808 |
| Detach | 3.19 | 0 | 585 | 6.37 | 4.19 | 6 | 1418 |
| Beggar | 3.22 | 0 | 682 | 6.53 | 4.91 | 6 | 36 |
| Frigid | 3.5 | 0 | 297 | 5.69 | 4.75 | 6 | 758 |
| Ordeal | 3.77 | 0 | 1131 | 7.03 | 4.92 | 6 | 1922 |
| Period | 3.8 | 0 | 53751 | 10.89 | 4.3 | 6 | 1958 |
| Dead | 1.94 | 10 | 72864 | 11.2 | 5.73 | 4 | 588 |
| Slum | 2.39 | 10 | 467 | 6.15 | 4.78 | 4 | 401 |
| Jail | 1.95 | 11 | 9659 | 9.18 | 5.49 | 4 | 236 |
| Slow | 3.93 | 13 | 43719 | 10.69 | 3.39 | 4 | 982 |
| Rape | 1.25 | 14 | 12684 | 9.45 | 6.81 | 4 | 344 |
| Fail | 1.79 | 15 | 18678 | 9.84 | 6.31 | 4 | 1531 |
| Fake | 3.1 | 15 | 7853 | 8.97 | 5.26 | 4 | 1535 |
| Hate | 2.12 | 16 | 44130 | 10.69 | 6.95 | 4 | 201 |
| Lame | 3.66 | 16 | 7593 | 8.93 | 3.69 | 4 | 1768 |
| Wart | 2.41 | 19 | 224 | 5.41 | 4.34 | 4 | 2438 |
| Pale | 3.17 | 21 | 5424 | 8.6 | 3.5 | 4 | 1935 |
| Crime | 2.89 | 5 | 33496 | 10.42 | 5.41 | 5 | 704 |
| Bored | 2.95 | 5 | 7024 | 8.86 | 2.83 | 5 | 48 |
| Waste | 2.93 | 5 | 27081 | 10.21 | 4.14 | 5 | 485 |
| Whore | 2.3 | 6 | 2885 | 7.97 | 5.85 | 5 | 492 |
| Louse | 2.81 | 6 | 100 | 4.61 | 4.98 | 5 | 262 |
| Stink | 3 | 6 | 1542 | 7.34 | 4.26 | 5 | 411 |
| Slave | 1.84 | 6 | 13149 | 9.48 | 6.21 | 5 | 398 |
| Shame | 2.13 | 7 | 11304 | 9.33 | 6.33 | 5 | 2167 |
| Bitch | 3 | 9 | 9406 | 9.15 | 6.36 | 5 | 1182 |
| Grave | 2.18 | 9 | 7527 | 8.93 | 4.78 | 5 | 1642 |
| Scare | 3.62 | 9 | 4294 | 8.36 | 6.56 | 5 | 2137 |
| Cancer | 1.5 | 5 | 18210 | 9.81 | 6.42 | 6 | 60 |
| Shamed | 2.5 | 5 | 221 | 5.4 | 4.88 | 6 | 386 |
| Mangle | 3.9 | 6 | 301 | 5.71 | 5.44 | 6 | 861 |
| Bicker | 3.21 | 6 | 154 | 5.04 | 5.36 | 6 | 1176 |
| Hooker | 3.34 | 6 | 1095 | 7 | 4.93 | 6 | 793 |
| Wounds | 2.51 | 6 | 2743 | 7.92 | 5.82 | 6 | 620 |
| Pester | 2.77 | 7 | 239 | 5.48 | 5.77 | 6 | 1963 |
| Hinder | 3.81 | 7 | 908 | 6.81 | 4.12 | 6 | 790 |
| Scared | 2.78 | 7 | 7739 | 8.95 | 6.82 | 6 | 604 |
| Wicked | 2.96 | 8 | 4211 | 8.35 | 6.09 | 6 | 493 |
