# Supplementary material for: A Controlled Approach to the Emotional Dilution of the Stroop Effect
Source: PLoS One. 2013 Nov 6;8(11):e80141. doi: 10.1371/journal.pone.0080141 (PMC3819280; doi:10.1371/journal.pone.0080141)
Supplement: Table S2 — Neutral-matched word characteristics. The neutral-matched words individual data for the valence, neighbourhood density, word frequency, arousal, and length. Also show is the ANEW code as per the ANEW guidelines set by Bradley & Lang (2010) [29]. Organised according to neighbourhood density followed by length of the word (zero to high neighbourhood; short to long). (DOCX) [file pone.0080141.s002.docx]

**Table S2.**

|  |  |  | Word frequency | |  |  |  |
| --- | --- | --- | --- | --- | --- | --- | --- |
| Words | Valence | Neighbourhood density | HAL norms | LOG transform | Arousal | Length | ANEW code |
| Echo | 6.17 | 0 | 29638 | 10.3 | 5.07 | 4 | 1470 |
| Hymn | 6.13 | 0 | 2288 | 7.74 | 4.33 | 4 | 1707 |
| Idol | 6.12 | 0 | 1920 | 7.56 | 4.95 | 4 | 802 |
| Rely | 5.27 | 0 | 8349 | 9.03 | 4.66 | 4 | 2074 |
| Adult | 6.49 | 0 | 25223 | 10.14 | 4.76 | 5 | 546 |
| Arena | 6.4 | 0 | 10812 | 9.29 | 6.43 | 5 | 1101 |
| Erupt | 5.43 | 0 | 395 | 5.98 | 6.57 | 5 | 1504 |
| Arrow | 5.17 | 0 | 7324 | 8.9 | 5.37 | 5 | 1105 |
| Radar | 4.54 | 0 | 7554 | 8.93 | 5.57 | 5 | 2041 |
| Entry | 5.69 | 0 | 34588 | 10.45 | 4.72 | 5 | 1495 |
| Cliff | 4.67 | 0 | 5132 | 8.54 | 6.25 | 5 | 553 |
| Chalk | 4.89 | 0 | 1662 | 7.42 | 3.48 | 5 | 1268 |
| Await | 4.64 | 0 | 1682 | 7.43 | 4.54 | 5 | 1125 |
| Wheel | 5.55 | 0 | 19368 | 9.87 | 4.86 | 5 | 2451 |
| Album | 6.21 | 0 | 39796 | 10.59 | 5.18 | 5 | 1073 |
| Thigh | 5.77 | 0 | 2526 | 7.83 | 5.47 | 5 | 2338 |
| Relic | 5.76 | 0 | 4915 | 8.5 | 5.16 | 5 | 2071 |
| Bugle | 5.27 | 0 | 435 | 6.08 | 4.73 | 5 | 1226 |
| Doctor | 5.2 | 0 | 31917 | 10.37 | 5.86 | 6 | 129 |
| Window | 5.91 | 0 | 54926 | 10.91 | 3.97 | 6 | 495 |
| Speech | 4.54 | 0 | 36422 | 10.5 | 5.68 | 6 | 2245 |
| Tissue | 4.93 | 0 | 14342 | 9.57 | 3.63 | 6 | 2358 |
| Frenzy | 4.97 | 0 | 2055 | 7.63 | 6.86 | 6 | 1589 |
| Salute | 5.92 | 0 | 1321 | 7.19 | 5.31 | 6 | 370 |
| Tattoo | 5.62 | 0 | 3407 | 8.13 | 6.06 | 6 | 2319 |
| Mascot | 6.29 | 0 | 611 | 6.42 | 5.1 | 6 | 1828 |
| Silent | 4.79 | 0 | 9257 | 9.13 | 3.46 | 6 | 2188 |
| Soccer | 5.9 | 0 | 7502 | 8.92 | 5.39 | 6 | 2230 |
| Squash | 4.97 | 0 | 1217 | 7.1 | 3.93 | 6 | 2256 |
| Voyage | 6.25 | 0 | 2283 | 7.73 | 5.55 | 6 | 1028 |
| Icebox | 4.95 | 0 | 40 | 3.69 | 4.17 | 6 | 799 |
| Belong | 6.46 | 0 | 14806 | 9.6 | 5.64 | 6 | 1170 |
| Wild | 6.37 | 8 | 23869 | 10.08 | 6.43 | 4 | 2452 |
| Doll | 6.09 | 11 | 5685 | 8.65 | 4.24 | 4 | 728 |
| Seal | 5.81 | 15 | 6421 | 8.77 | 5.42 | 4 | 2152 |
| Bake | 6.17 | 17 | 2528 | 7.84 | 5.1 | 4 | 647 |
| Lane | 5.39 | 18 | 14139 | 9.56 | 4.64 | 4 | 1770 |
| Bear | 4.78 | 19 | 23529 | 10.07 | 6.52 | 4 | 1159 |
| Dare | 5.76 | 19 | 8885 | 9.09 | 6.57 | 4 | 1391 |
| Mile | 5.24 | 21 | 9657 | 9.18 | 4.69 | 4 | 1849 |
| Male | 5.84 | 23 | 58587 | 10.98 | 4.9 | 4 | 1816 |
| Core | 4.8 | 23 | 19755 | 9.89 | 4.18 | 4 | 1347 |
| Plane | 6.43 | 5 | 18677 | 9.84 | 6.14 | 5 | 539 |
| Poker | 6.31 | 5 | 4519 | 8.42 | 5.64 | 5 | 1989 |
| Crave | 4.88 | 5 | 694 | 6.54 | 6.13 | 5 | 1364 |
| Chase | 5.07 | 5 | 9066 | 9.11 | 6.5 | 5 | 1273 |
| Naked | 6.34 | 6 | 13367 | 9.5 | 5.8 | 5 | 892 |
| Ducks | 6.2 | 6 | 2221 | 7.71 | 4.34 | 5 | 1455 |
| Stove | 4.98 | 7 | 1846 | 7.52 | 4.51 | 5 | 1001 |
| Spice | 6.21 | 7 | 2561 | 7.85 | 5.62 | 5 | 2247 |
| Diver | 6.45 | 7 | 1734 | 7.46 | 5.04 | 5 | 510 |
| Night | 6.06 | 7 | 97524 | 11.49 | 5.94 | 5 | 1890 |
| Grass | 6.12 | 8 | 7354 | 8.9 | 4.14 | 5 | 768 |
| Spine | 5.12 | 8 | 5167 | 8.55 | 4.48 | 5 | 2249 |
| Beads | 5.54 | 9 | 2210 | 7.7 | 4.11 | 5 | 1157 |
| Rower | 5.31 | 10 | 157 | 5.06 | 4.97 | 5 | 2114 |
| Store | 5.93 | 13 | 53126 | 10.88 | 5 | 5 | 2272 |
| Puddle | 5.07 | 6 | 909 | 6.81 | 4.14 | 6 | 2028 |
| Ballet | 5.61 | 6 | 1669 | 7.42 | 4 | 6 | 1139 |
| Gender | 5.73 | 6 | 11021 | 9.31 | 4.38 | 6 | 763 |
| Picket | 4.97 | 6 | 850 | 6.75 | 4.45 | 6 | 1965 |
| Render | 4.5 | 7 | 4183 | 8.34 | 4.65 | 6 | 2077 |
| Wander | 5.27 | 7 | 2521 | 7.83 | 4.93 | 6 | 2431 |
| Humble | 5.86 | 7 | 5095 | 8.54 | 3.74 | 6 | 219 |
