# Supplementary material for: A Controlled Approach to the Emotional Dilution of the Stroop Effect
Source: PLoS One. 2013 Nov 6;8(11):e80141. doi: 10.1371/journal.pone.0080141 (PMC3819280; doi:10.1371/journal.pone.0080141)
Supplement: Table S3 — Neutral-nomatched word characteristics. The neutral-nonmatched words individual data for the valence, neighbourhood density, word frequency, arousal, and length. Also show is the ANEW code as per the ANEW guidelines set by Bradley & Lang (2010) [29]. Organised according to the length of words (short to long). (DOCX) [file pone.0080141.s003.docx]

**Table S3.**

|  |  |  | Word frequency | |  |  |  |
| --- | --- | --- | --- | --- | --- | --- | --- |
| Words | Valence | Neighbourhood density | HAL Norms | LOG transform | Arousal | Length | ANEW code |
| Oil | 4.6 | 3 | 34785 | 10.46 | 4.72 | 3 | 1911 |
| Ice | 4.8 | 14 | 117269 | 11.67 | 4.83 | 3 | 1709 |
| Kid | 5.46 | 20 | 11748 | 9.37 | 4.1 | 3 | 1757 |
| Net | 5.92 | 4 | 38056 | 10.55 | 4.67 | 3 | 1887 |
| Hat | 6.42 | 8 | 20833 | 9.94 | 5.35 | 3 | 783 |
| Road | 4.37 | 11 | 6661 | 8.8 | 4.59 | 4 | 2102 |
| Wool | 4.39 | 17 | 173 | 5.15 | 4.54 | 4 | 2465 |
| Mule | 4.43 | 8 | 15887 | 9.67 | 5.3 | 4 | 1875 |
| Vice | 4.76 | 13 | 91869 | 11.43 | 4.18 | 4 | 2418 |
| Punk | 4.8 | 23 | 19755 | 9.89 | 4.18 | 4 | 2033 |
| Rock | 5.15 | 7 | 1488 | 7.31 | 4.27 | 4 | 965 |
| Wall | 5.27 | 6 | 2066 | 7.63 | 3.6 | 4 | 2430 |
| Deed | 5.35 | 14 | 3575 | 8.18 | 3.97 | 4 | 1399 |
| Bean | 5.4 | 18 | 512 | 6.24 | 4.33 | 4 | 1158 |
| Wane | 5.53 | 6 | 66722 | 11.11 | 4.9 | 4 | 2432 |
| Mane | 5.56 | 12 | 44285 | 10.7 | 4.52 | 4 | 1819 |
| Core | 5.85 | 13 | 1981 | 7.59 | 5.15 | 4 | 1347 |
| Robot | 4.11 | 0 | 6475 | 8.78 | 3.95 | 5 | 2106 |
| Habit | 4.48 | 3 | 3011 | 8.01 | 4.91 | 5 | 775 |
| Dwarf | 4.83 | 4 | 600 | 6.4 | 3.23 | 5 | 1463 |
| Thumb | 4.95 | 2 | 28116 | 10.24 | 3.79 | 5 | 2351 |
| Diary | 5.11 | 0 | 4768 | 8.47 | 4.32 | 5 | 1423 |
| Apple | 5.31 | 5 | 3379 | 8.13 | 4.97 | 5 | 1095 |
| Metal | 5.36 | 0 | 10880 | 9.29 | 4.71 | 5 | 874 |
| Novel | 5.39 | 3 | 2761 | 7.92 | 5.43 | 5 | 1897 |
| Beard | 5.52 | 1 | 6586 | 8.79 | 4.54 | 5 | 1160 |
| Alley | 5.61 | 3 | 1616 | 7.39 | 4.74 | 5 | 637 |
| Clown | 5.64 | 3 | 826 | 6.72 | 3.46 | 5 | 1311 |
| Manor | 5.73 | 1 | 2683 | 7.89 | 3.69 | 5 | 1820 |
| Scarf | 5.82 | 3 | 3173 | 8.06 | 4.3 | 5 | 2138 |
| Spoon | 5.93 | 4 | 2624 | 7.87 | 4.15 | 5 | 2251 |
| Broom | 6.07 | 2 | 12250 | 9.41 | 4.61 | 5 | 1218 |
| Tread | 6.41 | 2 | 65844 | 11.1 | 4.17 | 5 | 2380 |
| Right | 6.45 | 7 | 388384 | 12.87 | 4.79 | 5 | 2097 |
| System | 4.5 | 1 | 47846 | 10.78 | 4.3 | 6 | 2311 |
| Column | 4.9 | 0 | 1396 | 7.24 | 4.34 | 6 | 695 |
| Museum | 5 | 0 | 76 | 4.33 | 4.64 | 6 | 889 |
| Galaxy | 5.09 | 2 | 1272 | 7.15 | 4.41 | 6 | 1609 |
| Breast | 5.17 | 0 | 15765 | 9.67 | 3.62 | 6 | 51 |
| Suburb | 5.17 | 1 | 7801 | 8.96 | 3.46 | 6 | 2289 |
| Satire | 5.21 | 3 | 5258 | 8.57 | 4.57 | 6 | 2134 |
| Enzyme | 5.37 | 0 | 439448 | 12.99 | 3.93 | 6 | 1498 |
| Pulpit | 5.39 | 1 | 12193 | 9.41 | 5 | 6 | 2030 |
| Rebuff | 5.43 | 1 | 1435 | 7.27 | 3.9 | 6 | 2054 |
| Policy | 5.45 | 0 | 371 | 5.92 | 4.11 | 6 | 1992 |
| Hockey | 5.5 | 4 | 3830 | 8.25 | 4.2 | 6 | 1693 |
| Statue | 5.54 | 0 | 10047 | 9.22 | 3.6 | 6 | 995 |
| Tennis | 5.62 | 2 | 44083 | 10.69 | 4.93 | 6 | 540 |
| Canyon | 5.76 | 1 | 4250 | 8.35 | 5.29 | 6 | 1245 |
| Retire | 5.89 | 0 | 1845 | 7.52 | 4.54 | 6 | 2087 |
| Carrot | 5.93 | 3 | 1311 | 7.18 | 5.2 | 6 | 1253 |
| Status | 6.02 | 1 | 6412 | 8.77 | 4.61 | 6 | 2262 |
| Empire | 6.03 | 5 | 51799 | 10.86 | 5 | 6 | 1483 |
| Breath | 6.07 | 2 | 14432 | 9.58 | 4.77 | 6 | 1212 |
| Saucer | 6.21 | 0 | 9295 | 9.14 | 5.96 | 6 | 2136 |
| Closet | 6.23 | 0 | 1641 | 7.4 | 5.34 | 6 | 1306 |
| Riddle | 6.26 | 2 | 16228 | 9.69 | 5.65 | 6 | 2096 |
| Cattle | 6.37 | 1 | 2307 | 7.74 | 4.7 | 6 | 1260 |
| Wonder | 6.5 | 0 | 7545 | 8.93 | 5.37 | 6 | 499 |
| Thought | 4.76 | 0 | 99256 | 11.51 | 4.71 | 7 | 1013 |
| Science | 4.82 | 0 | 7701 | 8.95 | 3.93 | 7 | 2143 |
| Stomach | 5.93 | 2 | 8546 | 9.05 | 4.36 | 7 | 998 |
| Cockpit | 6.31 | 0 | 2318 | 7.75 | 5.53 | 7 | 1316 |
| Glasses | 6.39 | 0 | 174954 | 12.07 | 4.83 | 7 | 1626 |
